# Supplementary material for: Iterative improvement in the automatic modular design of robot swarms
Source: PeerJ Comput Sci. 2020 Dec 7;6:e322. doi: 10.7717/peerj-cs.322 (PMC7924708; doi:10.7717/peerj-cs.322)
Supplement: Supplemental Information 3 [file peerj-cs-06-322-s003.zip › argos3/doc/api/standalone/a00628_source.html]

ARGoS: plugins/simulator/physics\_engines/pointmass3d/pointmass3d\_quadrotor\_model.h Source File


- Main Page
- Related Pages
- Namespaces
- Classes
- Files

- File List
- File Members

# plugins/simulator/physics\_engines/pointmass3d/pointmass3d\_quadrotor\_model.h

Go to the documentation of this file.

```
00001 
00007 #ifndef POINTMASS3D_QUADROTOR_H
00008 #define POINTMASS3D_QUADROTOR_H
00009 
00010 namespace argos {
00011    class CPointMass3DEngine;
00012    class CPointMass3DQuadRotorModel;
00013    class CQuadRotorEntity;
00014 }
00015 
00016 #include <argos3/plugins/simulator/physics_engines/pointmass3d/pointmass3d_model.h>
00017 #include <argos3/core/simulator/entity/embodied_entity.h>
00018 #include <argos3/plugins/simulator/entities/quadrotor_entity.h>
00019 
00020 namespace argos {
00021 
00022    class CPointMass3DQuadRotorModel : public CPointMass3DModel {
00023 
00024    public:
00025       
00026       CPointMass3DQuadRotorModel(CPointMass3DEngine& c_engine,
00027                                  CEmbodiedEntity& c_body,
00028                                  CQuadRotorEntity& c_quadrotor,
00029                                  Real f_body_height,
00030                                  Real f_arm_length,
00031                                  Real f_body_mass,
00032                                  Real f_body_inertia,
00033                                  const CVector3& c_pos_kp,
00034                                  const CVector3& c_pos_kd,
00035                                  Real f_yaw_kp,
00036                                  Real f_yaw_kd,
00037                                  const CVector3& c_vel_kp,
00038                                  const CVector3& c_vel_kd,
00039                                  Real f_rot_kp,
00040                                  Real f_rot_kd,
00041                                  const CVector3& c_max_force = CVector3(1000.0f, 1000.0f, 1000.0f),
00042                                  Real f_max_torque = 1000.0f);
00043 
00044       virtual ~CPointMass3DQuadRotorModel() {}
00045       
00046       virtual void Reset();
00047 
00048       virtual void UpdateFromEntityStatus();
00049       virtual void Step();
00050 
00051       virtual void CalculateBoundingBox();
00052 
00053       virtual bool CheckIntersectionWithRay(Real& f_t_on_ray,
00054                                             const CRay3& c_ray) const;
00055 
00056       virtual void UpdateOriginAnchor(SAnchor& s_anchor);
00057 
00058    private:
00059 
00060       void PositionalControl();
00061       void SpeedControl();
00062 
00063       Real PDControl(Real f_cur_error,
00064                      Real f_k_p,
00065                      Real f_k_d,
00066                      Real& f_old_error);
00067    private:
00068 
00070       CQuadRotorEntity& m_cQuadRotorEntity;
00071 
00073       Real m_fBodyHeight;
00074 
00076       Real m_fArmLength;
00077 
00079       Real m_fBodyMass;
00080 
00082       Real m_fBodyInertia;
00083 
00085       CVector3 m_cPosKP;
00086 
00088       CVector3 m_cPosKD;
00089       
00091       Real m_fYawKP;
00092 
00094       Real m_fYawKD;
00095 
00097       CVector3 m_cVelKP;
00098 
00100       CVector3 m_cVelKD;
00101 
00103       Real m_fRotKP;
00104 
00106       Real m_fRotKD;
00107 
00109       CVector3 m_cMaxForce;
00110 
00112       Real m_fMaxTorque;
00113 
00115       CQuadRotorEntity::SPositionControlData m_sDesiredPositionData;
00116 
00118       CQuadRotorEntity::SSpeedControlData m_sDesiredSpeedData;
00119 
00121       CRadians m_cYaw;
00122 
00124       CRadians m_cRotSpeed;
00125 
00127       CRadians m_cTorque;
00128 
00130       CVector3 m_cLinearControl;
00131 
00133       Real m_fRotationalControl;
00134 
00136       Real m_pfLinearError[3];
00137 
00139       Real m_fRotError;
00140    };
00141 
00142 }
00143 
00144 #endif
```

---

Generated on 10 Jul 2018 for ARGoS by 
 1.6.1
